# Highly Pathogenic Avian Influenza Virus A/H5N1 Subclade 2.3.4.4b Isolated from a European Grey Seal (*Halichoerus grypus*) Is Highly Virulent in Ferrets

**DOI:** 10.1093/infdis/jiaf348

**Published:** 2025-06-28

**Authors:** Kate Guilfoyle, Monica Mirolo, Leon de Waal, Geert van Amerongen, Guido van der Net, Theresa Störk, Mara Sophie Lombardo, Wolfgang Baumgärtner, Ásgeir Bjarnason, Hekla Bryndis Jóhannsdóttir, Guus Rimmelzwaan, Martin Ludlow, Albert Osterhaus

**Affiliations:** Preclinical Speciality Services, Cerba Research (Formerly Viroclinics Xplore), Schaijk, The Netherlands; Research Center for Emerging Infections and Zoonoses, University of Veterinary Medicine, Hannover, Germany; Preclinical Speciality Services, Cerba Research (Formerly Viroclinics Xplore), Schaijk, The Netherlands; Preclinical Speciality Services, Cerba Research (Formerly Viroclinics Xplore), Schaijk, The Netherlands; Preclinical Speciality Services, Cerba Research (Formerly Viroclinics Xplore), Schaijk, The Netherlands; Department of Pathology, University of Veterinary Medicine, Hannover, Germany; Department of Pathology, University of Veterinary Medicine, Hannover, Germany; Department of Pathology, University of Veterinary Medicine, Hannover, Germany; Star-Oddi, Ltd, Gardabaer, Iceland; Star-Oddi, Ltd, Gardabaer, Iceland; Research Center for Emerging Infections and Zoonoses, University of Veterinary Medicine, Hannover, Germany; Research Center for Emerging Infections and Zoonoses, University of Veterinary Medicine, Hannover, Germany; Research Center for Emerging Infections and Zoonoses, University of Veterinary Medicine, Hannover, Germany

**Keywords:** HPAI, H5N1, intratracheal, pathogenicity, ferrets

## Abstract

Highly pathogenic avian influenza A viruses subtype H5N1 (HPAIV H5N1), subclade 2.3.4.4b infect multiple avian and mammalian species, posing a potential pandemic risk. Here we describe the outcomes of infection of ferrets with an HPAIV H5N1 virus, isolated from a European grey seal in 2023, compared with an older HPAIV H5N1 (A/Indonesia/05/2005). Overall, infection of ferrets with A/grey seal/Netherlands/302603/2023 caused more rapid mortality than infection of ferrets with A/Indonesia/05/2005. Animals developed severe pneumonia and irreversible hypothermia, associated with high levels of virus replication and histopathological changes in the respiratory tract and peripheral organs. As animal models for severe avian influenza virus infections in humans play a key role in the development of intervention strategies against these infections, these findings highlight the importance of using updated ferret models based on circulating virus strains.

## Teaser

Intratracheal infection with a H5N1 2.3.4.4b strain caused accelerated mortality in ferrets, characterized by severe pneumonia and irreversible hypothermia prior to death or euthanasia, associated with high levels of virus replication and histopathological changes in respiratory tract, liver, and spleen.

Almost 30 years have passed since the detection of a highly pathogenic avian influenza (HPAI) virus subtype H5N1, named A/Goose/Guangdong/1/96 (GsGd) H5N1, in Southern China in 1996 [1]. GsGd H5N1 caused outbreaks in poultry, human cases in Hong Kong in 1997 [2, 3], and subsequently spread to many regions of Southeastern Asia, including Vietnam, Indonesia, and Thailand, and later to West Asia and North Africa. Widespread circulation in birds has led to genetic and antigenic diversification due to antigenic drift and shift with cocirculating low pathogenic avian influenza (LPAI) viruses in wild bird species [4]. GsGd H5N1 strains are categorized into 10 antigenic clades (0 to 9), each with further subgroup divisions [4, 5]. Clade 2 is divided into 5 subgroups (2.1 to 2.5), with further subdivisions. Human infection with clade 2.1 Indonesian viruses caused 160 reported fatal cases between 2005 and 2012 [6], and infections with H5N1 clade 2.2 in Egypt resulted in 116 reported human deaths between 2005 and 2015 due to severe pneumonia [7]. From 2021, the predominantly circulating influenza A/H5Nx in Europe and the Americas belonged to subclade 2.3.4.4b [8]. This subclade was first detected in Eurasian wigeons (*Mareca penelope*) in the Netherlands in October 2020 with genome analyses showing that this strain was a reassortant characterized by HA and MP gene segments deriving from an Iraqi-like H5N8 strain and all remaining gene segments originating from circulating Eurasian avian lineage LPAI A/H5Nx strains [9]. These strains have since spread within Europe and later to Africa, Asia, and the Americas, predominantly by migratory birds [10]. Reported human infections with H5N1 decreased substantially in recent years compared to the period between 2005 and 2015. H5N1 subclade 2.3.4.4b strains have caused more than 100 reported infections in humans in China, Chile, Ecuador, Spain, United Kingdom, and the United States from 2021 [8, 11, 12]. In the same period, the H5N1 clade 2.3.4.4b strains have displayed increased transmissibility in animals compared to older GsGd H5N1 strains. This has resulted in an unprecedented epizootic among avian and mammalian species. The H5N1 clade 2.3.4.4b strains circulating from November 2021 have remained epizootic in wild birds, with multiple isolated infections of mammal species reported in all continents except Oceania, and most recently in fur seals (*Arctocephalus gazella**)*** in Antarctica's islands [13], neonatal goats (*Capra hircus*) and dairy cows (*Bos taurus*) in the United States [14, 15]. Recent sporadic infections with H5N1 subclade 2.3.4.4b genotype B3.13 caused mild disease in dairy farm workers following reported contact with infected cows or contaminated milk. Infections with other reassorted H5N1 2.3.4.4b strains continue to cause severe pneumonia and mortality in humans [16]. The severe pneumonia induced by some H5N1 strains in humans can be recapitulated by intratracheal inoculation of ferrets (*Mustela putorius furo*) and cynomolgus macaques (*Macaca fascicularis*) [17–20]. The rapid evolution of H5N1 strains through reassortment events remains a concern and underscores the critical role of animal models in testing the efficacy of preventive and therapeutic intervention strategies for H5N1 human infections. In the present study, we describe clinical, virological, and pathological findings following infection of ferrets with an H5N1 clade 2.3.4.4b strain isolated from a European grey seal (*Halichoerus grypus*) in 2023 [21]. Our study highlights differences in mortality of ferrets infected with a contemporary H5N1 strain, compared to a previously frequently used H5N1 A/Indonesia/05/2005 virus. This study provides an updated animal model for the development and testing of preventive and therapeutic interventions for H5N1 clade 2.3.4.4b infections in humans.

## METHODS

### Viruses

The input virus strains used in this infection experiment were HPAI H5N1 A/grey seal/Netherlands/302603/2023 clade 2.3.4.4b virus and HPAI H5N1 A/Indonesia/5/2005 clade 2.1.3.2. Further details are provided in Supplementary Material, Section i. All virus handling was performed under biosafety level 3 (BSL-3) conditions.

### Experimental Protocol

**Figure 1.**
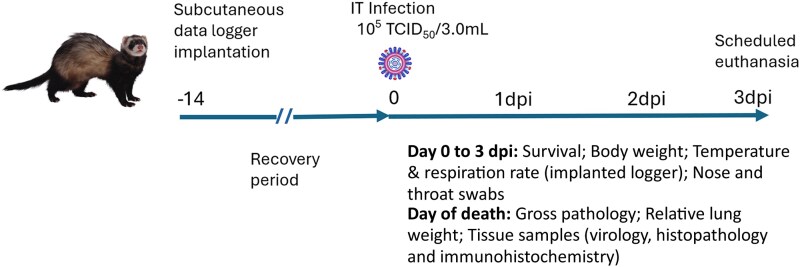
Experimental design and timeline. A subcutaneous data logger was implanted in ferrets. After a 14-day recovery period, the animals were infected IT with either H5N1 A/grey seal/NL/2023 clade 2.3.4.4b or H5N1 A/Indo/2005 clade 2.1.3.2 virus. The animals were followed for a period of 3 dpi, the scheduled end point of the experiment. Abbreviations: dpi, days postinfection; IT, intratracheally; TCID_50_, 50% tissue culture infectious dose.

Nine 13-month-old female ferrets (Euroferrets, Denmark; animal Nos 1 to 9), were shown to be negative for antibodies against currently circulating strains of influenza virus by hemagglutination inhibition assay as described previously [22]. The experimental design and time line is illustrated in Figure 1. Data loggers (DST micro-ACT data logger, diameter 8.3 mm, length 24.4 mm, weight 3.3 g; Star-Oddi) were subcutaneously implanted in ferrets. Data logger information is provided in Supplementary Material, Section ii. Following a 2-week recovery period, preinfection throat and nose swabs were collected from all animals. Subsequently, ferrets were infected with 10^5.0^ 50% tissue culture infectious dose (TCID_50_) A/grey seal/NL/2023 (ferret Nos 1 to 6) or 10^5.0^ TCID_50_ A/Indo/2005 (ferret Nos 7 to 9) intratracheally using a total volume of 3.0 mL. The chosen route, dose, and volume were based on previous experimental infections of ferrets with A/Indonesia/5/2005 virus [17, 19, 23, 24]. Animals were monitored daily for clinical symptoms, weighed, and throat and nose swabs were collected. At 3 days postinfection (dpi), animals were euthanized (by abdominal exsanguination under ketamine anesthesia) and necropsies were performed. During necropsy, data loggers were retrieved, animals were examined for gross pathological changes, and lungs were collected, observed for gross lesions, and weighed. Relative lung weight (RLW) was calculated using individual lung weight/body weight (time of death) × 100. Tissue samples were collected from lungs, nasal turbinate, spleen, liver, and brain (olfactory bulb, cerebrum, and cerebellum) and stored at −80°C for virological analyses, and for histopathological and immunohistochemical analyses fixed in 10% buffered formal saline for slide preparation. If an animal reached a predefined humane end point (unresponsive, respiratory distress) before 3 dpi, it was euthanized, examined, and samples were collected as described above. The experiment was performed according to Dutch and European regulations and was registered under project license AVD27700202216449-WP3, written following ARRIVE (Animal Research: Reporting of In Vivo Experiments) guidelines.

### Titration of Swab and Tissue Samples

Tissue homogenate supernatant and swabs collected were titrated in MDCK cell monolayers, seeded in 96-well tissue culture plates to achieve approximately 90% confluence. Four 10-fold serial dilutions (10^−1^ to 10^−7^) of each sample were prepared in 96-well dilution plates using infection medium. Medium composition and assay method is provided in Supplementary Material, Section iii. Five days after infection, virus titers were evaluated by a hemagglutination assay using turkey erythrocytes (0.33% v/v). Virus titers were calculated using the Spearman-Karber method and normalized for tissue sample weight (gram) or swab volume (mL). The lower limit of detection (LLOD) for the throat and nose swabs was 0.8 log_10_ TCID_50_/mL. The LLOD for the tissue samples was dependent on individual sample weight (log_10_ TCID_50_/g). Statistical analyses were performed using Mann-Whitney in Graphpad Prism (version 5.0) software. Differences were considered significant at *P* < .05.

### RT-qPCR of Swab and Tissue Samples

Total RNA was extracted from 140 µL of sample in lysis buffer using QIAamp Viral RNA Mini Kit protocol (Qiagen) according to manufacturer instructions and in an elution volume of 30 μL. The RNA was tested by reverse transcription quantitative polymerase chain reaction (RT-qPCR) using primers that amplify a 95-bp region of the influenza A virus matrix gene (M) [21]. To enable quantification of detectable viral RNA in the samples collected postinfection, the number of viral RNA copies was calculated from the positive control standard curve established during validation of the RT-qPCR assay (Supplementary Material, Section iv). Individual tissue sample weight was also included in the calculation (log_10_ copies/g). Statistical analyses were performed using Mann-Whitney in Graphpad Prism (version 5.0) software. Differences were considered significant at *P* < .05.

### Histopathology and Immunohistochemistry

Formalin-fixed tissue samples (lung, trachea, nose, liver, spleen, kidneys, adrenals, brain, pancreas, and intestine) of 9 ferrets (animal Nos 1 to 9) were obtained for histological and immune-histological examination. After dehydration, samples were embedded in paraffin and 2-µm sections were cut and stained with hematoxylin and eosin (H&E). Additionally, immunohistochemistry (IHC) was performed for each organ with influenza A nucleoprotein (AIV NP) staining using anti-influenza A antibody HB65, mouse, monoclonal (Biozol Diagnostics Vertrieb, GmbH). Evaluation and scoring scales for tissues are provided in Supplementary Material, Section v and Section vi.

### Next-Generation Sequencing and Bioinformatic Analyses

Whole-genome sequencing analyses was performed on total RNA extracted from the stocks of A/grey seal/NL/2023 and A/Indo/5/2005, and from supernatant from lung homogenates of four ferrets (Nos 1, 2, 8, and 9). Extraction details are provided in Supplementary Material, Section vii. Raw reads were assembled to the respective references A/grey seal/Netherlands/302603/2023, EPI_ISL_17672782 and A/Indonesia/05/2005, EPI_ISL_5729. Full-genome sequences were compiled and analyzed for variants using QIAGEN CLC Genomics Workbench (version 12).

## RESULTS

### Clinical Evaluation

**Figure 2.**
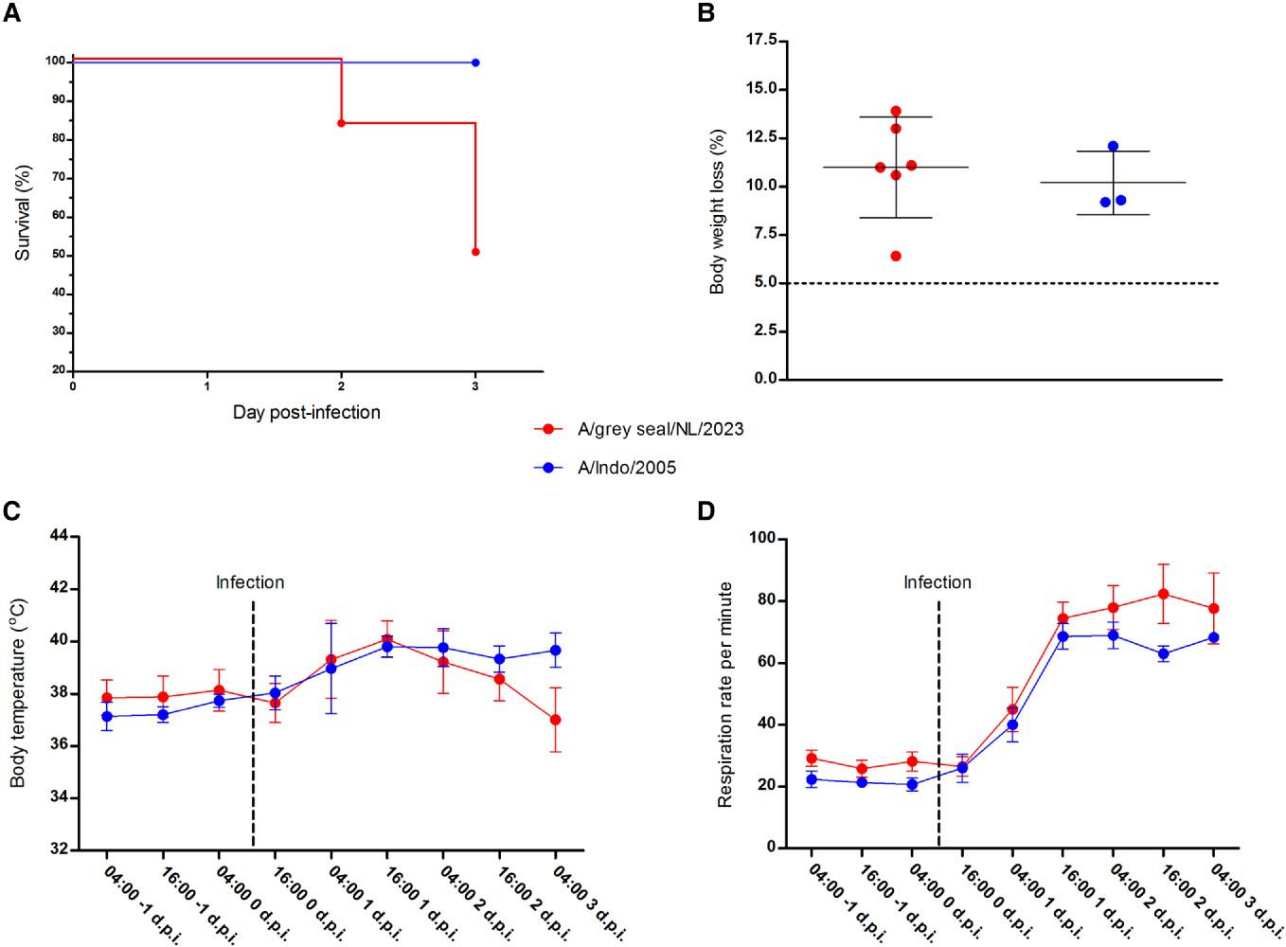
Clinical parameters of virus-infected ferrets. *A*, Percent survival to 3 days postinfection (dpi). *B*, Percent body weight loss from day 0 (before infection) to 3 dpi. Body weight changes < 5% are considered within normal weight fluctuations, 5% is indicated by a horizontal dashed line. *C*, Body temperature (°C). *D*, Respiration rate per minute. All data are group mean ± SD. Clinical parameters are presented for ferrets infected with A/grey seal/NL/2023 clade 2.3.4.4b or A/Indo/2005 clade 2.1.3.2 viruses.

Low survival rates were observed for ferrets infected with A/grey seal/NL/2023 (Nos 1 to 6) (Figure 2*A*). Ferret No. 2 had to be euthanized at 2 dpi, as it had reached the predefined humane end point. Two other ferrets (Nos 1 and 5) were found dead in the morning of day 3, prior to the scheduled euthanasia. The remaining 3 animals infected with A/grey seal/NL/2023 (Nos 3, 4, and 6), and 3 ferrets infected with A/Indo/2005 (Nos 7, 8, and 9), survived until the scheduled time of euthanasia (3 dpi). The latter is also in agreement with previous ferret infection studies with A/Indo/2005, in which no animals had succumbed to the infection before the scheduled time of euthanasia [17, 19, 23 , 24]. All ferrets infected in this study displayed clinical signs indicative of severe AIV infection, including breathing difficulties, lethargy, and weight loss. At 3 dpi, body weight loss ranged between 6.4% and 13.9%, with no significant difference between the respective infected groups (Figure 2*B*). Neurological signs were not observed in any of the ferrets. Body temperatures peaked at 1 dpi up to 41°C and progressively declined below basal levels (approximately 38°C) at 3 dpi in ferrets infected with A/grey seal/NL/2023, while remaining at peak levels for A/Indo/2005-infected ferrets until 3 dpi (Figure 2*C*). A general increase of respiration rates was reported for all 9 animals until 1 dpi, after which values kept increasing until time of death for 4 animals infected with A/grey seal/NL/2023 (Nos 1, 2, 4, and 5). By 3 dpi, respiration rate had stabilized in the remaining 2 animals infected with A/grey seal/NL/2023 and animals infected with A/Indo/2005 (Figure 2*D*, Supplementary Table 1, and Supplementary Figure 2).

### Infectivity Titration and RT-qPCR

**Figure 3.**
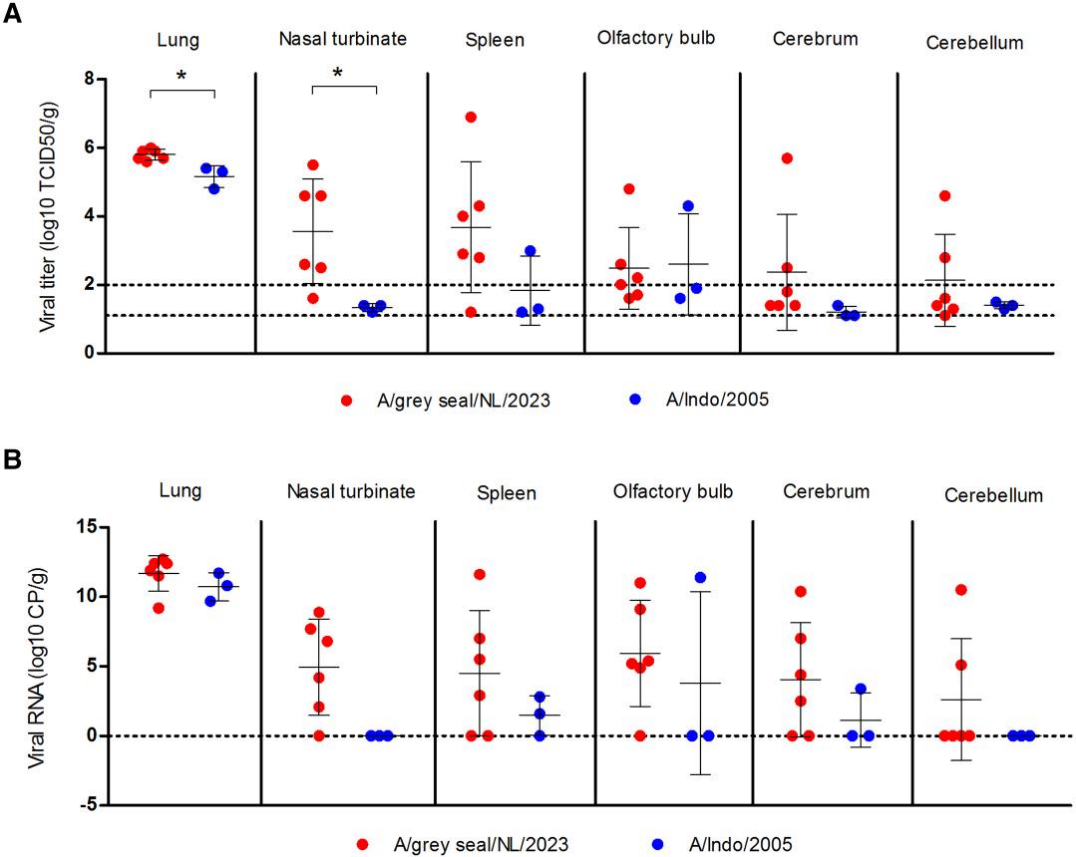
Virological analyses of tissue samples from virus-infected ferrets: (*A*) levels of infectious virus (log_10_ TCID_50_/g) and (*B*) viral RNA (log_10_ copies/g) from lung, nasal turbinate, spleen, olfactory bulb, cerebrum, and cerebellum tissue samples collected from ferrets infected with either H5N1 A/grey seal/NL/2023 or A/Indo/2005 viruses. Individual values are shown with group mean ± SD (solid black lines). LLOD for the titration assay was variable as calculated using the individual tissue sample weight; the LLOD range is indicated by dashed lines (*A*). Samples with an undetermined cycle threshold value were assigned a value of zero, indicated by a dashed line (*B*). *P* values were calculated by Mann-Whitney test. *P < .05. Abbreviations: LLOD, lower limit of detection; TCID_50_, 50% tissue culture infectious dose.

Virus titration and RT-qPCR were performed on throat and nose swabs, lung, nasal turbinate, spleen, olfactory bulb, cerebrum, and cerebellum. Mean A/grey seal/NL/2023 titers in lungs and nasal turbinates were significantly higher than those found in the respective samples from the A/Indo/2005-infected ferrets and, although individually not significant, there was a tendency towards higher titers in the spleen, cerebrum, and cerebellum in these animals (Figure 3*A*). Overall, mean levels of A/grey seal/NL/2023 RNA copies were also higher than A/Indo/2005 RNA copies for all analyzed tissue samples, but again the differences were not significant (Figure 3*B*).

**Figure 4.**
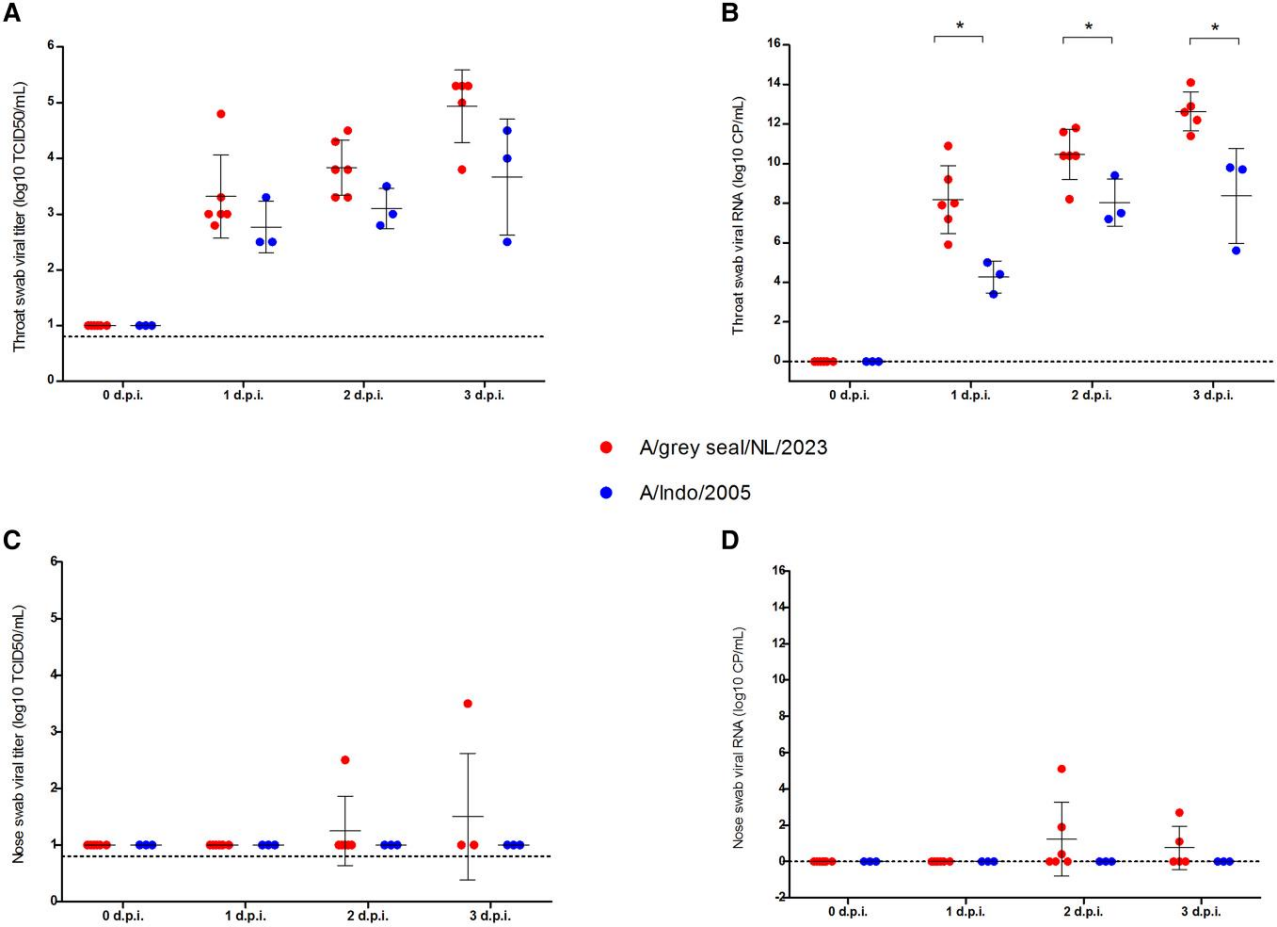
Virological analyses of throat and nose swabs from ferrets. Levels of infectious virus (log_10_ TCID_50_/mL) in (*A*) throat and (*C*) nose swabs of ferrets infected with either A/grey seal/NL/2023 or A/Indo/2005 viruses from day 0 (before infection) to 3 dpi. Levels of viral RNA (log_10_ copies/mL) in (*B*) throat and (*D*) nose swabs of ferrets infected with A/grey seal/NL/2023 (red) and A/Indo/2005 (blue) viruses from day 0 (before infection) to 3 dpi. Individual values are shown with group mean ± SD (solid black lines). Lower limit of detection (0.8 TCID_50_/mL) for the titration is indicated by a dashed line (*A* and *C*). Samples with an undetermined cycle threshold value were assigned a value of zero, indicated by a dashed line (*B* and *D*). *P* values were calculated by Mann-Whitney test. *P < .02. Abbreviation: TCID_50_, 50% tissue culture infectious dose.

The mean infectious titers in the throats of A/grey seal/NL/2023-infected ferrets were higher than those infected with A/Indo/2005 (Figure 4*A*). The A/grey seal/NL/2023 RNA copies from throat swabs of ferrets were significantly higher than those of A/Indo/2005 throughout the experiment, (Figure 4*B*). In the nose swabs from A/grey seal/NL/2023-infected ferrets, little or no infectious virus (Figure 4*C*) or RNA copies (Figure 4*D*) were detected. No evidence of A/Indo/2005 viral replication in the nose was found by titration or RT-qPCR.

### Gross Pathology of Ferret Lungs

**Figure 5.**
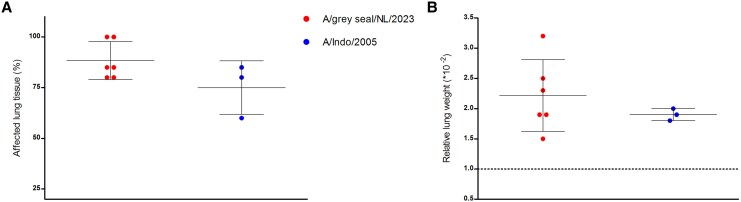
Gross-pathology of virus-infected ferrets. *A*, Observed percentage affected lung tissue at the time of necropsy. *B*, Relative lung weight (calculated as individual lung weight/body weight × 100). Lungs with a RLW ≤ 1.0 are considered healthy or slightly pneumonic, RLW 1.0 is indicated by a horizontal dashed line. Individual values are shown with group mean ± SD (solid black lines).

At necropsy, whole lungs were extracted, weighed, and the percentage of observed affected lung tissue was recorded to estimate the extent and severity of lung lesions. All 9 ferrets displayed comparable levels of gross pathological changes in lungs (Figure 4*A*), comprising multifocal and diffuse dark red areas of consolidation. Darker lung tissue was observed in lungs of ferrets Nos 1 and 5 infected with A/grey seal/NL/2023. These 2 animals died overnight between 2 and 3 dpi and necropsy was performed several hours post mortem together with ferrets euthanized as per the experimental protocol. For these 2 animals, the percentage of affected tissue was estimated at 100%, while for all other animals the percentage of affected lung tissue was between 60% and 80% (Figure 5*A*).

An RLW value less than or equal to 1 is indicative of healthy or slightly pneumonic lungs. The RLW was >1.0 for all 9 animals, indicative of severe lung inflammation. Mean RLW value was slightly higher for ferrets infected with A/grey seal/NL/2023 (mean RLW = 2.2) than for those infected with A/Indo/2005 (mean RLW = 1.9) (Figure 5*B*).

### Histopathology and Immunohistochemistry

**Figure 6.**
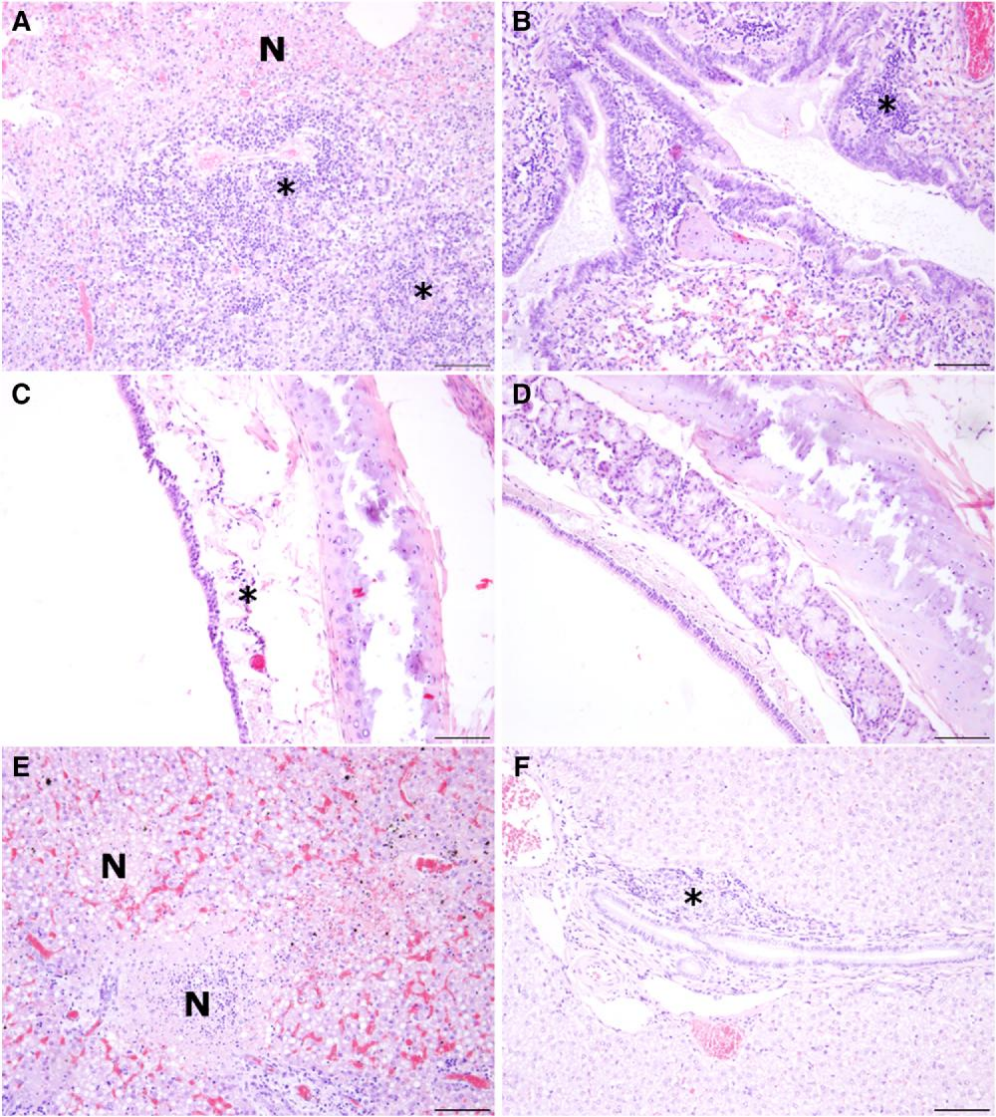
Inflammatory changes within lung, trachea, and liver of virus-infected ferrets. *A*, Lung necrosis (N) and multifocal, lymphohistiocytic, partly neutrophilic, perivascularly accentuated infiltrates in ferret No. 5 (*). *B*, Peribronchial cuffing in ferret No. 8 (*). *C*, Mild lymphohistiocytic inflammation within the trachea of ferret No. 1 (*). *D*, No infiltrates within the trachea in ferret No. 8. *E*, Oligofocal to multifocal areas of necrosis (N) in the liver of ferret No. 1. *F*, Mild lymphohistiocytic pericholangitis (*) within the liver of ferret No. 8. Hematoxylin and eosin staining, scale bar: 100 µm.

Histological findings are summarized in Supplementary Table 2 and consisted mainly of alveolar damage, intra-alveolar inflammatory cells, and peribronchial cuffing within the lungs of all animals (Figure 6*A* and 6*B*). Mild tracheitis was found in 3 out of 6 animals infected with A/grey seal/NL/2023 (Figure 6*C*), while other animals showed no infiltrates within the trachea (Figure 6*D*). Within the nose, no major histopathological changes were observed. Only ferret No. 1 infected with A/grey seal/NL/2023 virus displayed moderate inflammation in respiratory and olfactory mucosa (H&E score 1–2; Supplementary Table 2).

Extrapulmonary organs, including liver and spleen, presented major histopathological changes for ferret Nos 1 and 5 infected with A/grey seal/NL/2023 virus, which were found dead at 3 dpi. The liver and the spleen of these animals presented moderate to severe necrosis (Figure 6*E*) and inflammatory changes (Figure 6*F*).

**Figure 7.**
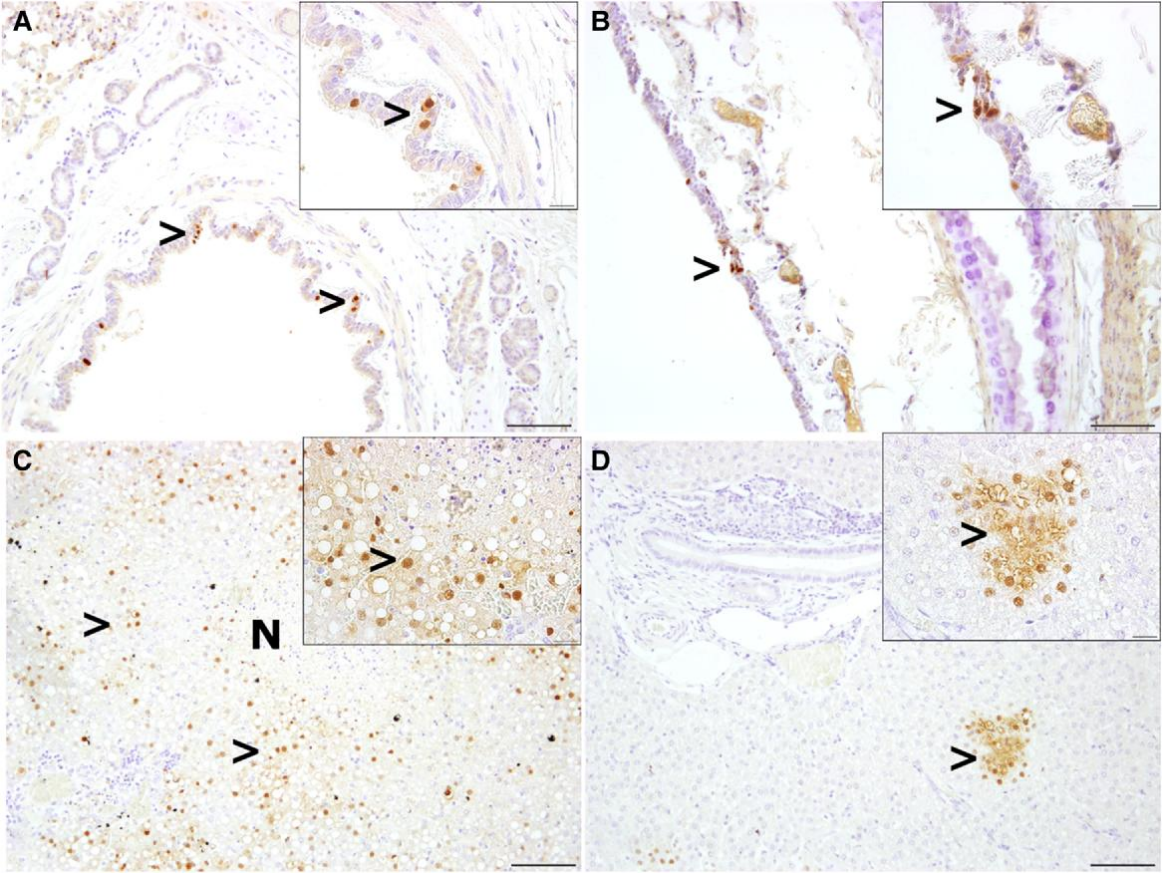
Detection of influenza A nucleoprotein (AIV NP) antigen in lung, trachea, and liver of virus-infected ferrets. *A*, AIV NP antigen in single bronchial epithelial cells in lung tissue in ferret No. 1 (>). *B*, AIV NP antigen in single or small clusters of cells within the trachea epithelium of ferret No. 1 (>). *C*, Moderate to high AIV NP antigen (>) expression in hepatocytes and lung necrosis (N) of ferret No. 1. *D*, Focal AIV NP expression in hepatocytes in ferret No. 8. Inserts show immunopositive cells at higher magnification. Immunohistochemistry, anti-influenza A antibody HB65; scale bar, 100 µm; scale bar insert, 20 µm.

Equal levels of histopathological changes or lack of lesions were present in the brain, kidney, pancreas, intestine, and adrenals. Lesions consisted primarily of minor lymphohistiocytic infiltrates and/or necrosis (Supplementary Table 2). The immunohistochemical findings are summarized in Supplementary Table 3. Intralesional AIV NP antigen was found in varying degrees within the epithelial cells of bronchi, bronchioles, and alveoli (Figure 7*A*). The trachea showed a limited AIV NP expression in 4 of 6 animals infected with A/grey seal/NL/2023 (Figure 7*B*), and no AIV NP expression in the trachea of ferrets infected with A/Indo/2005. In ferret No. 1, moderate to high numbers of hepatocytes and low to moderate numbers of cells in the spleen contained AIV NP antigen (IHC score 2–3) (Figure 7*C*), whereas the other animals showed no or very mild AIV NP antigen signal within liver and spleen (IHC score 0–1) (Figure 7*D*). Within brain, nose, and kidneys, single AIV NP immunolabeled cells were observed in single animals infected with A/grey seal/NL/2023 (IHC score 1; Supplementary Table 3). Viral antigen was not detected in any animal of both groups within the intestine, pancreas, and adrenals (IHC score 0; Supplementary Table 3).

### Next-Generation Sequencing Analyses of HPAI Viruses

The genetic diversity of the individual HPAIV H5N1 gene segments and their closest relatives are provided in Supplementary Table 4. The consensus genome sequences of A/grey seal/NL/2023 and A/Indo/2005 input viruses, both passaged twice in MDCK cells prior to use in this experiment, were identical to the original respective virus stocks A/grey seal/Netherlands/302603/2023 (EPI_ISL_17672782) and A/Indonesia/5/2005 (EPI_ISL_5729). A/grey seal/NL/2023 did not possess the amino acid substitutions E627K, D701N, and S714R in the PB2 protein that promote replication in mammals, nor mutations Q226L and G228S in the HA protein that increase binding of the H5 with alpha 2,6-linked sialic acid receptors. A lysine was found at position 339 of PB2 (PB2-339K) of A/grey seal/NL/2023, not present in A/Indo/2005 virus. The amino acid K at position 339 of the grey seal H5N1 virus (PB2-339K) was present in 100% of reads of the input virus and 100% of reads of viruses from the lung and no low-frequency variants were detected at this level. Metagenomic sequencing of total RNA extracted from the lung homogenate samples of 2 animals infected with A/grey seal/NL/2023 showed that the virus did not acquire other mutations in the consensus genome during the 3-day experiment.

## DISCUSSION

The panzootic spread of avian H5N1 subclade 2.3.4.4b among birds and mammals, its rapid evolution through reassortment with LPAI viruses, its expansion of geographical range, and its spread into progressively increasing numbers of terrestrial and marine mammals has raised concerns with respect to the increasing pandemic risk. Experimental H5N1 infection of cows [25] has shown that the North American scenario of widespread H5N1 genotype B3.13 infections in cows with sporadic infections in dairy farm workers might also occur in other geographical areas where this virus is enzootic [26]. Therefore, we selected for our infection experiments in ferrets A/grey seal/NL/2023 as a representative of circulating H5N1 subclade 2.3.4.4b in Europe: the H5N1 strain belongs to EA-2021-AB (H5N1 A/duck/Saratov/29-02/2021-like), one of the dominant A/H5 genotypes circulating in avian species in Europe [8].

In this experiment we investigated the pathogenesis and virulence of A/grey seal/NL/2023 virus infection in ferrets and compared this to outcomes from ferrets infected, in the same experiment, with A/Indo/2005 virus. These data were supported by historical published data from previous ferret infection experiments using the same A/Indo/2005 virus [17, 19, 23, 24].

Three of the 6 ferrets infected with A/grey seal/NL/2023 had to be euthanized or succumbed as a result of a severe multisystemic disease course. Death before 3 dpi has not been previously observed in ferrets infected intratracheally with 10^5^ TCID_50_ of A/Indo/2005 but consistently reported from 4 dpi [17, 19, 23, 24]. Death of ferrets prior to 4 dpi with A/Indonesia/5/2005 virus has been previously reported, but this was following infection with a higher concentration of the virus [18].

A recent risk assessment study evaluating the pathogenicity and transmissibility of the first-reported human-infecting bovine H5N1 virus, A/Texas/37/2024 genotype B3.13 in ferrets, reported death of animals within 3 days, in contrast to experimental infections of ferrets with H5N1 clade 2.3.4.4b viruses A/American Wigeon/SC/22–000345–001/2021 (bird isolate), A/dairy cow/Texas/24-008749-001/2024 (dairy cattle isolate), A/Chile/25945/2023 (human isolate), and A/mink/Spain/ 22VIR12774-13_3869-2/2022 (mink, generated by reverse genetics), which caused death of ferrets between 4 and 10 dpi [27–31]. In contrast to the present study, the aforementioned experimental infections were done with H5N1 viruses assigned to genotypes other than A/grey seal/NL/2023, including B3.13, A1/EA-2020-C, B3.13, B3.2, and EA-2022-BB for A/Texas/37/2024, A/American Wigeon/SC/22-000345-001/2021, A/dairy cow/Texas/24-008749-001/2024, A/Chile/25945/2023, and A/mink/Spain/ 22VIR12774-13_3869-2/2022 respectively. Additionally, experimental infection of ferrets with the aforementioned viruses was performed via the intranasal route using a different concentration of virus.

Virological investigations showed that A/grey seal/NL/2023 replicated to significantly higher levels than A/Indo/2005 in ferret nasal turbinate and lung tissue. The A/grey seal/NL/2023 was also more abundantly present in the pharynx than A/Indo/2005 throughout the 3-day period. The presence of a lysine at position 339 of PB2 (PB2-339K) of A/grey seal/NL/2023, previously associated with increased replication in lungs and virulence in mice [32], which was not present in A/Indo/2005 viruses, may have played a role by enhancing replication in the ferret lungs. No mutations in A/grey seal/NL/2023 were acquired during the relatively short experiment. In contrast to differences in survival rates and limited differences in the virological data, similar levels of inflammation and necrosis were observed in the lung, nose, caudal, and apical part of the trachea of all 9 ferrets by H&E and IHC. Conflicting results between virological and pathological analyses may pertain to a lower sensitivity of fluorescence in situ hybridization (FISH) for formalin-fixed paraffin-embedded (FFPE) tissues [33] and/or the bias derived from the possible under-representative number of FFPE sections evaluated (1 for each lung lobe, and caudal/apical part of trachea, nose), given the focal localization of virus replication sites. It is interesting to note that in contrast to the A/grey seal/NL/2023-infected ferrets, no evidence of A/Indo/2005 replication in the nose of ferrets was found.

None of the ferrets displayed overt neurological symptoms, as has previously been reported in published literature on A/Indo/2005 intratracheal infections in the ferret model [17]. However, in our study intratracheal infection with A/grey seal/NL/2023 or A/Indo/2005 did result in low-level virus replication in the ferret olfactory bulb. Mild histopathological changes were detected in only 1 of the virologically positive ferrets inoculated with A/grey seal/NL/2023, with AIV NP antigen detected in neurons by IHC staining (ferret No. 4). Brain lesions, often not described in H5N1 ferret models for pneumonia using A/Indo/2005 virus [17, 18], were previously found in cats and foxes upon H5N1 intratracheal inoculation, probably contributing to fatal outcome of infection in these animals [34, 35].

It has been well documented in mammalian species that H5N1 viruses primarily infect the respiratory tract when inoculated intratracheally [17]; however, lethal pathogenesis is also associated with virus replication in extrarespiratory organs [36]. In our study, ferrets infected with A/grey seal/NL/2023 displayed severe inflammation of liver and spleen that was characterized by a mild to moderate inflammation and diffuse areas of necrosis, with AIV NP antigen present in necrotic hepatocytes and splenocytes. Although virological analyses revealed slightly higher mean levels of replicating virus and RNA copies in the spleen of ferrets infected with A/grey seal/NL/2023 than in the ferrets infected with A/Indo/2005, these differences were not statistically significant.

Hypothermia and tachypnoea were detected prior to the death of 3 A/grey seal/NL/2023-infected ferrets. In contrast, high yet stable body temperature and respiration rates were observed in ferrets infected with A/Indo/2005, corroborating previously published results in which all ferrets survived until at least 4 dpi with this virus [17, 19, 23, 24].

In conclusion, our data show that intratracheal infection of ferrets with A/grey seal/NL/2023 causes accelerated mortality, as compared to intratracheal infection with A/Indonesia/05/2005. Collectively, our data support the development and use of updated ferret models to test preventive and therapeutic intervention strategies for human H5N1 infections.

## Supplementary Material

jiaf348_Supplementary_Data

## Supplementary Data

Supplementary materials are available at *The Journal of Infectious Diseases* online (http://jid.oxfordjournals.org/). Supplementary materials consist of data provided by the author that are published to benefit the reader. The posted materials are not copyedited. The contents of all supplementary data are the sole responsibility of the authors. Questions or messages regarding errors should be addressed to the author.

## Notes

***Acknowledgments.*** The authors thank Kristin Laudeley for technical assistance, and the authors and submitting laboratories of sequences obtained from the GISAID's EpiFlu Database (http://www.gisaid.org). We also thank colleagues at Cerba Research, Schaijk for their technical and biotechnical assistance with experimental preparation as well as animal care and procedures.

***Author contributions.*** K. G., M. M., L. d. W., G. v. A., W. B., and A. O. designed the study. K. G., M. M., L. d. W., G. v. A., G. v. d. N., T. S., and M. S. L. performed the research. K. G., M. M., L. d. W., G. v. d. N., T. S., M. S. L., A. B., and H. B. J. analyzed the data. K. G. and M. M. wrote the draft manuscript. W. B, G. R., M. L., and A. O. edited the manuscript. All authors reviewed and approved the final manuscript.

***Financial support.*** The molecular virology work performed by M. M. was supported in part by the Deutsche Forschungsgemeinschaft (German Research Foundation; grant number 398066876/GRK 2485/2) as part of her PhD programme.
