# Supplementary material for: Highly Pathogenic Avian Influenza Virus A/H5N1 Subclade 2.3.4.4b Isolated from a European Grey Seal (Halichoerus grypus) Is Highly Virulent in Ferrets
Source: J Infect Dis. 2025 Jun 28;232(6):e886–96. doi: 10.1093/infdis/jiaf348 (PMC12718051; doi:10.1093/infdis/jiaf348)
Supplement: jiaf348_Supplementary_Data [file jiaf348_supplementary_data.docx]

**Supplementary Appendix**

1. **Virus and cell culture information……………………………………………...……....2**
2. **Data logger visual inspection and caluclation of respiratory rates……………………2**

Table S1 Number of respiration rate measurements per animal……………………….…………..3

Figure S1 Raw accelerometry data for animal #2……………………………………….………..3

Figure S2 Respiration rate and body temperature from each animal during the experiment……....4

1. **Infection medium composition and titration method….……………………………...4**
2. **Validation of an IAV RT-qPCR assay…………………………………….…………….5**

Figure S3. Amplification efficiency for the Influenza A MP-gene RT-qPCR assay……………….6

1. **Pathohistological scoring criteria and data..…………………………………………..6**

Table S2. Pathohistological findings by organ tissue type……………………………..……..…..7

1. **Immunohistochemical scoring criteria and data………………..……………...….....10**

Table S3. Immunohistochemical findings by organ tissue type………………...….………..…..11

1. **Protein sequence homology data…………………………………...………………….13**

Table S4. Protein sequence homology of A/grey seal/Netherlands/302603/2023 genome segments………………………………………………………………………………………...13

**Supplementary appendix references…………………..……………………………..…………...14**

**Alt Text Supplementary Figures and Tables……………………………………………………...15**

1. **Virus and cell culture information**

The HPAI H5N1 clade 2.3.4.4b virus A/grey seal/Netherlands/302603/2023 (EPI_ISL_17672782) previously propagated in Madin Derby canine kidney (MDCK) cells following isolation from the brain tissue homogenate of an adult grey seal as described previously [1]. was passaged once in MDCK (ATCC, USA) cells to produce the virus stock (passage 2) used as input virus “A/grey seal/NL/2023”. The full-length concatenated A/grey seal/NL/2023 genome sequence was most closely related to avian H5N1 genotype EA-2021-AB (H5N1 A/duck/Saratov/29-02/2021-like viruses), circulating in Germany and The Netherlands since November 2021 [2]. The H5N1 A/Indonesia/5/2005 clade 2.1.3.2 virus (EPI_ISL_5729) had been isolated from a human patient in Indonesia in 2005 and was also propagated in MDCK cells (passage 2) to produce the input virus stock “A/Indo/2005”. Input stocks were produced using infection medium (EMEM (Sigma; Cat#: M4655) supplemented with 2% HEPES (Gibco; Cat#: 15630-056), 0.3% Bovine Serum Albumin (Sigma; Cat#: A7979), 0.05% Trypsin-EDTA (Gibco; Cat#: 25300-62).

1. **Visual inspection and calculation of respiratory rates**

Data loggers were pre-programmed to measure body temperature every five minutes. Each day from 4 a.m. to 5 a.m. and from 4 p.m. to 5 p.m., data loggers recorded raw tri axial acceleration data at 10Hz for one minute. The respiration rate was derived from the tri axial acceleration data. We visually inspected and calculated respiration rate successfully from 379 raw 3-axis acceleration measurements recorded at 10Hz for 1 minute (Figure S1). Measurements were selected that had low average activity levels (AvgEA) of 6-10mg, indicating rest. The AvgEA calculation of activity provided by the manufacturer gives the average acceleration above standard gravity, which is defined with a static calibration, normalisation and finally a calculation of the vectoral sum in milli-g (mg) a method like calculation of the vectoral sum of body acceleration VeDBA [3] and minimum specific acceleration MSA [2]. Out of the three axes (X, Y and Z), the axis with the highest amplitude was selected as it can change based on the orientation of the animal and the logger. Peaks in the sinusoidal respiratory signal were annotated and respirations per minute calculated. Supplementary Figure S2 shows example for baseline signal -1 d.p.i. for animal #2 (logger 224) (A), maximum respiratory rate signal for animal #2 (logger 224) (B) and a 10 second period of that signal highlighting the high rate (C).

**Table S1.** Number of respiration rate measurements per animal

| **Animal** | **Virus** | **Logger ID** | **Number of successful RR measurements** |
| --- | --- | --- | --- |
| 1 | A/grey seal/NL/2023 | 222 | 30 |
| 2 |  | 224 | 35 |
| 3 |  | 225 | 54 |
| 4 |  | 229 | 40 |
| 5 |  | 226 | 44 |
| 6 |  | 227 | 57 |
| 7 | A/Indo/2005 | 230 | 47 |
| 8 |  | 223 | 27 |
| 9 |  | 228 | 45 |
|  | | **Total** | **379** |


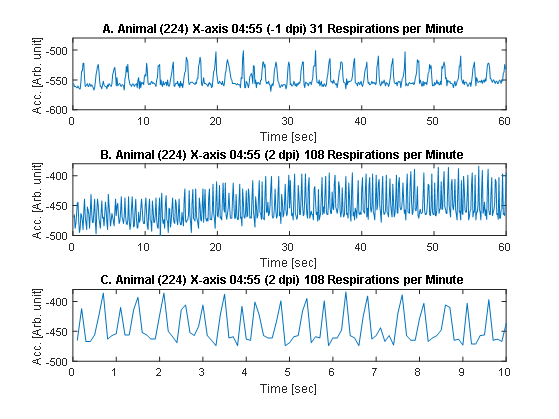
**Figure S1.** **Raw accelerometry data for animal #2**. (A) Raw accelerometry data from X-axis of animal #2 (implanted with logger 224) recorded from 04:55-04:56 (-1 dpi) calculated as 31 respirations per minute. (B) animal #2, 72 hours later with 108 Respirations per minute. (C) Shows the same signal as B but for the last ten seconds only.

F**igure S2**. Respiration rate (blue lines) and body temperature (orange lines) from each animal during the experiment. Data are depicted as mean ±SD for each animal. Asterix (*) is used to indicate ferrets that died prior to scheduled experimental endpoint (3 dpi).

1. **Infection medium composition and titration method**

Infection medium was comprised of (EMEM (Sigma; Cat#: M4655) supplemented with 2% HEPES (Gibco; Cat#: 15630-056), 0.3% Bovine Serum Albumin (Sigma; Cat#: A7979), 1% amphotericin B (Sigma; Cat#: A2942), 1% penicillin/streptomycin (Sigma; Cat#: P0781), 0.05% Trypsin-EDTA (Gibco; Cat#: 25300-62). MDCK cells monolayers were washed once with phosphate buffered saline (PBS) (Sigma; Cat#: D8537) and once with infection medium. Sample dilutions were directly transferred onto the washed cell monolayers (100 µL per well) and incubated for 90 minutes at 37°C, 5% CO2. The inoculum was removed, monolayers were washed twice with infection medium, and fresh infection medium was added prior to incubation at 37°C, 5% CO2.

1. **Validation of an IAV RT-qPCR assay**

Positive-control RNA was generated *in vitro* transcription of a cloned 95-bp fragment of Influenza A virus and quantified as previously described [4]. RNA was extracted using the QIAamp Viral RNA Mini Kit (Qiagen) according to manufacturer instructions. RNA was quantified using the RNA HS assay (Thermo Fisher Scientific, USA), and RNA integrity was assessed by estimation of the RIN value with an Agilent RNA 6000 Pico Kit (Agilent) in an Agilent Bioanalyzer 2200 (Agilent). Removal of ribosomal RNA, and metagenomic sequencing were performed on the high-throughput sequencing NovaSeq™ X Plus platform (Illumina) with 12Gb raw output per sample at Novogene Tech. The RNA that was generated from positive control was tested in the RT-qPCR assay using published primers [5] . All RT-qPCRs were performed using the Luna universal probe one-step RT-qPCR kit (NEB) using a concentration of 0.4 μM for each primer and 0.2 μM for the probe (FAM-TTTGTGTTCACGCTCACCGTGCC-TAMRA) in the RT-qPCR master mix. Assays were performed using a using a LightCycler 96 Real-Time PCR System (Roche) with an initial 10-min RT step at 55°C, followed by incubation for 1 min at 95°C. Forty-five two-step cycles were then performed using the following conditions: denaturation at 95°C for 10 s, annealing and amplification at 60°C for 30 s. The fluorescence level was detected and quantified. To produce the standard curves, the RNA from the positive control was diluted 1000-fold to reach a concentration of 1.9 x 10^10^ RNA copies/uL and then serially diluted 10 times. Four replicates of each dilution were tested in the RT-qPCR assay. The Cq values for each dilution were plotted against the respective log RNA concentrations and simple linear regression analysis was performed using GraphPad Prism 8 software to automatically produce slope, intercept, and R^2^ values. The resulting Cq values were plotted against the logarithm of each respective RNA concentration. The standard curve for the positive control showed a slope of -2.871 and a R^2^ of 0.9796 (Figure S4).

**Figure S3.** Amplification efficiency for the Influenza A MP-gene RT-qPCR assay previously designed [23]. Mean input RNA copy numbers/μl from ten replicates of 10-fold dilutions are plotted against the respective mean Cq values. The linear regression area of the plot is shown. Linear regression analysis was performed using GraphPad Prism 8 (GraphPad Software Inc.).

1. **Pathohistochemical scoring criteria and data**

Evaluation of HE-stained sections for liver, kidney, spleen, intestine, pancreas, adrenals and brain was done with a semi-quantitative three-tier scoring scale (score 0 - 6), which includes degree of influenza-induced tissue necrosis and inflammation. For lung tissue, HE evaluation comprised the extent and severity of influenza-induced alveolitis and alveolar damage, presence of alveolar edema, haemorrhage, type II pneumocyte hyperplasia, severity of bronchitis, bronchiolitis, degree of perivascular and peribronchial cuffing and tracheitis (cumulative score: 0-21). The total score for the nose included inflammation and necrosis of respiratory mucosa, epithelial hyper- and/or metaplasia, inflammation and necrosis of olfactory mucosa, intraluminal exudate and vasculopathy (cumulative score: 0-23). Scoring system for severity of alveolitis, bronchitis, bronchiolitis and perivascular/peribronchial cuffing in the lung: 0 = no inflammation, 1 = mild inflammation, 2 = moderate inflammation, 3 = severe inflammation; Scoring system for extend of alveolitis in the lung: 0 = 0%, 1 = <25%, 2 = 25-50%, 3 = >50% of tissue affected; Scoring system for the presence of alveolar edema, alveolar hemorrhage and type II pneumocyte hyperplasia in the lung: 0 = no, 1 = yes; Scoring system for extent of necrosis and inflammation of respiratory mucosa and extent of necrosis and inflammation of olfactory mucosa in the nose: 0 = 0%, 1 = ≤1%, 2= 2-25%, 3 = 26-50%, 4 = 51-75%, 5 = > 75 % of tissue affected; Scoring system for the presence of epithelial hyper- and/or metaplasia of respiratory epithelium, intraluminal exudate and vasculopathy in the nose: 0 = no, 1 = yes; Scoring system for necrosis in liver, kidney, spleen, intestine, pancreas, adrenals and brain: 0 = no necrotic foci, 1 = <2 necrotic foci, 2 = 2-6 necrotic foci; 3 = >6 necrotic foci; Scoring system for inflammation in liver, kidney, spleen, intestine, pancreas, adrenals and brain and pericholangitis in the pancreas: 0 = no inflammation, 1 = mild inflammation, 2 = moderate inflammation, 3 = severe inflammation; - no animal displayed this score; * adrenals not available in animal #2.

**Table S2**. Pathohistological findings

| Organ | Lesion | Score | Number and ID of affected animals infected with | | | |
| --- | --- | --- | --- | --- | --- | --- |
|  |  |  | A/grey seal/NL/2023 virus (n=6) | | A/Indo/2005 virus (n=3) | |
| Lung | Extent of alveolitis/ alveolar damage | 0 | - | Median score:  2 | - | Median score:  2 |
|  |  | 1 | 1 (#2) |  | - |  |
|  |  | 2 | 4 (#1, #3, #4, #6) |  | 2 (#8, #9) |  |
|  |  | 3 | 1 (#5) |  | 1 (#7) |  |
|  | Severity of alveolitis | 0 | - | Median score:  3 | - | Median score:  3 |
|  |  | 1 | - |  | - |  |
|  |  | 2 | 2 (#1, #2) |  | 1 (#8) |  |
|  |  | 3 | 4 (#3-#6) |  | 2 (#7, #9) |  |
|  | Presence of alveolar edema | 0 | - | Median score:  1 | - | Median score:  1 |
|  |  | 1 | 6 (#1-#6) |  | 3 (#7-#9) |  |
|  | Presence of alveolar hemorrhage | 0 | - | Median score:  1 | - | Median score:  1 |
|  |  | 1 | 6 (#1-#6) |  | 3 (#7-#9) |  |
|  | Presence of type II pneumocyte hyperplasia | 0 | 1 (#1) | Median score:  1 | - | Median score:  1 |
|  |  | 1 | 5 (#2-#6) |  | 6 (#1-#6) |  |
|  | Severity of bronchitis | 0 | - | Median score:  1,5 | - | Median score:  2 |
|  |  | 1 | 3 (#1, #3, #4) |  | - |  |
|  |  | 2 | 2 (#2, #5) |  | 6 (#1-#6) |  |
|  |  | 3 | 1 (#6) |  | - |  |
|  | Severity of bronchiolitis | 0 | - | Median score:  2 | - | Median score:  2 |
|  |  | 1 | 2(#1, #2) |  | - |  |
|  |  | 2 | 3 (#3, #5, #6) |  | 6 (#1-#6) |  |
|  |  | 3 | 1 (#4) |  | - |  |
|  | Degree of peribronchial/ perivascular cuffing | 0 | - | Median score:  2 | - | Median score:  2 |
|  |  | 1 | 1 (#1) |  | 1 (#8) |  |
|  |  | 2 | 4 (#2-#4, #6) |  | 2 (#7, #9) |  |
|  |  | 3 | 1 (#5) |  |  |  |
| Trachea | Inflammation | 0 | 2 (#3, #6) | Median score:  1 | 1 (#9) | Median score:  1 |
|  |  | 1 | 4 (#1, #2, #4, #5) |  | 2 (#7, #8) |  |
|  |  | 2 | - |  | - |  |
|  |  | 3 | - |  | - |  |
| Median of total lung score including trachea (maximum attainable score: 21) | | | 14 | | 14 | |
| Nose | Extent of necrosis of respiratory mucosa | 0 | 2 (#5, #6) | Median score:  1 | - | Median score:  1 |
|  |  | 1 | 3 (#2-#4) |  | 3 (#7-#9) |  |
|  |  | 2 | 1 (#1) |  | - |  |
|  |  | 3 | - |  | - |  |
|  |  | 4 | - |  | - |  |
|  |  | 5 | - |  | - |  |
|  | Extent of inflammation of respiratory mucosa | 0 | - | Median score:  2 | - | Median score:  2 |
|  |  | 1 | 2 (#5, #6) |  | 1 (#7) |  |
|  |  | 2 | 3 (#2-#4) |  | 2 (#8, #9) |  |
|  |  | 3 | 1 (#1) |  | - |  |
|  |  | 4 | - |  | - |  |
|  |  | 5 | - |  | - |  |
|  | Presence of epithelial hyper- and/or metaplasia of respiratory epithelium | 0 | 1 (#5) | Median score:  1 | 2 (#7, #8) | Median score:  0 |
|  |  | 1 | 5 (#1-#4, #6) |  | 1 (#9) |  |
|  | Extent of necrosis of olfactory mucosa | 0 | 2 (#5, #6) | Median score:  1 | - | Median score:  1 |
|  |  | 1 | 3 (#2-#4) |  | 3 (#7-#9) |  |
|  |  | 2 | 1 (#1) |  | - |  |
|  |  | 3 | - |  | - |  |
|  |  | 4 | - |  | - |  |
|  |  | 5 | - |  | - |  |
|  | Extent of inflammation of olfactory mucosa | 0 | 1 (#6) | Median score:  1 | - | Median score:  1 |
|  |  | 1 | 4 (#2-#5) |  | 3 (#7-#9) |  |
|  |  | 2 | - |  | - |  |
|  |  | 3 | 1 (#1) |  | - |  |
|  |  | 4 | - |  | - |  |
|  |  | 5 | - |  | - |  |
|  | Presence of intraluminal exudate | 0 | 6 (#1-#6) | Median score:  0 | 3 (#7-#9) | Median score:  0 |
|  |  | 1 | - |  | - |  |
|  | Presence of vasculopathy | 0 | 4 (#2, #3, #5, #6) | Median score:  0 | 3 (#7-#9) | Median score:  0 |
|  |  | 1 | 2 (#1, #4) |  | - |  |
| Median of total nose score (maximum attainable score: 23) | | | 6 | | 5 | |
| Liver | Necrosis | 0 | 1 (#2) | Median score: 1,5 | 1 (#9) | Median score:  1 |
|  |  | 1 | 2 (#3, #6) |  | 2 (#7, #8) |  |
|  |  | 2 | 2 (#4, #5) |  | - |  |
|  |  | 3 | 1 (#1) |  | - |  |
|  | Inflammation | 0 | - | Median score: 2 | - | Median score:  1 |
|  |  | 1 | 2 (#1, #3) |  | 3 (#7-#9) |  |
|  |  | 2 | 4 (#2, #4, #5, #6) |  | - |  |
|  |  | 3 | - |  | - |  |
| Median of total liver score (maximum attainable score: 6) | | | 3 | | 2 | |
| Kidney | Necrosis | 0 | 6 (#1-#6) | Median score:  0 | 3 (#7-#9) | Median score:  0 |
|  |  | 1 | - |  | - |  |
|  |  | 2 | - |  | - |  |
|  |  | 3 | - |  | - |  |
|  | Inflammation | 0 | - | Median score:  1 | 1 (#7) | Median score:  1 |
|  |  | 1 | 5 (#1-#3, #5, #6) |  | 1 (#9) |  |
|  |  | 2 | 1 (#4) |  | 1 (#8) |  |
|  |  | 3 | - |  | - |  |
| Median of total kidney score (maximum attainable score: 6) | | | 1 | | 1 | |
| Spleen | Necrosis | 0 | 2 (#3, #4) | Median score:  1 | 2 (#8, #9) | Median score:  0 |
|  |  | 1 | 2 (#2, #6) |  | 1 (#7) |  |
|  |  | 2 | 2 (#1, #5) |  | - |  |
|  |  | 3 | - |  | - |  |
|  | Inflammation | 0 | - | Median score:  1 | - | Median score:  1 |
|  |  | 1 | 6 (#1-#6) |  | 3 (#7-#9) |  |
|  |  | 2 | - |  | - |  |
|  |  | 3 | - |  | - |  |
| Median of total spleen score (maximum attainable score: 6) | | | 2 | | 1 | |
| Intestine | Necrosis | 0 | 6 (#1-#6) | Median score:  0 | 3 (#7-#9) | Median score:  0 |
|  |  | 1 | - |  | - |  |
|  |  | 2 | - |  | - |  |
|  |  | 3 | - |  | - |  |
|  | Inflammation | 0 | - | Median score:  1 | - | Median score:  1 |
|  |  | 1 | 6 (#1-#6) |  | 2 (#7, #9) |  |
|  |  | 2 | - |  | 1 (#8) |  |
|  |  | 3 | - |  | - |  |
| Median of total intestine score (maximum attainable score: 6) | | | 1 | | 1 | |
| Pancreas | Necrosis | 0 | 3 (#2, #4, #5) | Median score:  0,5 | 2 (#7, #9) | Median score:  0 |
|  |  | 1 | 3 (#1, #3, #6) |  | 1 (#8) |  |
|  |  | 2 | - |  | - |  |
|  |  | 3 | - |  | - |  |
|  | Inflammation | 0 | 1 (#4) | Median score:  1 | 1 (#9) | Median score:  1 |
|  |  | 1 | 4 (#1, #3, #5, #6) |  | 1 (#7) |  |
|  |  | 2 | 1 (#2) |  | - |  |
|  |  | 3 | - |  | 1 (#8) |  |
|  | Perichol-angitis | 0 | 1 (#3) | Median score:  1 | - | Median score:  1 |
|  |  | 1 | 3 (#1, #4, #6) |  | 2 (#8, #9) |  |
|  |  | 2 | 2 (#2, #5) |  | 1 (#7) |  |
|  |  | 3 | - |  | - |  |
| Median of total pancreas score (maximum attainable score: 9) | | | 3 | | 3 | |
| Adrenals* | Necrosis | 0 | 5 (#1, #3-#6) | Median score:  0 | 3 (#7-#9) | Median score:  0 |
|  |  | 1 | - |  | - |  |
|  |  | 2 | - |  | - |  |
|  |  | 3 | - |  | - |  |
|  | Inflammation | 0 | 3 (#1, #3-#6) | Median score:  0 | 2 (#8, #9) | Median score:  0 |
|  |  | 1 | 2 (#1, #4) |  | 1 (#7) |  |
|  |  | 2 | - |  | - |  |
|  |  | 3 | - |  | - |  |
| Median of total adrenal score (maximum attainable score: 6) | | | 0 | | 0 | |
| Brain | Necrosis | 0 | 6 (#1-#6) | Median score:  0 | 3 (#7-#9) | Median score:  0 |
|  |  | 1 | - |  | - |  |
|  |  | 2 | - |  | - |  |
|  |  | 3 | - |  | - |  |
|  | Inflammation | 0 | 5 (#1-#4, #6) | Median score:  0 | 2 (#7, #9) | Median score:  0 |
|  |  | 1 | 1 (#5) |  | 1 (#8) |  |
|  |  | 2 | - |  | - |  |
|  |  | 3 | - |  | - |  |
| Median of total brain score (maximum attainable score: 6) | | | 0 | | 0 | |

1. **Immunohistochemical scoring criteria and data**

IHC evaluation was performed using a four-tier scoring system (score 0-4) for liver, kidney, spleen, intestine, pancreas, adrenal and brain. For nose and lung including trachea, a more detailed scoring scheme was applied (0-12). Scoring system for extent of immunopositive cells within alveolae and airway epithelium of the lung and within trachea, liver, kidney, spleen, intestine, pancreas, adrenals and brain: 0 = no positive cells, 1 = single positive cells, 2 = low numbers of positive cells, 3 = moderate numbers of positive cells, 4 = high numbers of positive cells; Scoring system for extent of immunopositive cells within respiratory and olfactory epithelium of the nose: 0 = no positive cells, 1 = ≤ 1% of epithelium positive, 2 = 2-25% of epithelium positive, 3 = 26-50% of epithelium positive, 4 = 51-75% of epithelium positive, 5 = > 75 % of epithelium positive; Scoring system for presence of immunopositive cells within the intraluminal exudate and immunopositive subepithelial cells in the nose: 0 = no, 1 = yes; - no animal displayed this score; * adrenals not available in animal #2.

**Table S3.** Immunohistological findings

| Organ | Analysed parameter | Score | Number and ID of affected animals infected with | | | |
| --- | --- | --- | --- | --- | --- | --- |
|  |  |  | A/grey seal/NL/2023 virus (n=6) | | A/Indo/2005 virus (n=3) | |
| Lung | Extent of immuno-positive cells within alveolae | 0 | - | Median score:  1 | - | Median score:  1 |
|  |  | 1 | 6 (#1-#6) |  | 3 (#7-#9) |  |
|  |  | 2 | - |  | - |  |
|  |  | 3 | - |  | - |  |
|  |  | 4 | - |  | - |  |
|  | Extent of immuno-positive cells within airway epithelium | 0 | - | Median score:  1 | 1 (#8) | Median score:  1 |
|  |  | 1 | 5 (#1-#5) |  | 2 (#7, #9) |  |
|  |  | 2 | 1 (#6) |  | - |  |
|  |  | 3 | - |  | - |  |
|  |  | 4 | - |  | - |  |
| Trachea | Extent of immuno-positive cells | 0 | 2 (#2, #6) | Median score:  1 | 3 (#7-#9) | Median score:  0 |
|  |  | 1 | 4 (#1, #3-#5) |  | - |  |
|  |  | 2 | - |  | - |  |
|  |  | 3 | - |  | - |  |
|  |  | 4 | - |  | - |  |
| Median of total IHC lung score including trachea (maximum attainable score: 12) | | | 3 | | 2 | |
| Nose | Extent of immuno-positive cells within respiratory epithelium | 0 | 6 (#1-#6) | Median score:  0 | 3 (#7-#9) | Median score:  0 |
|  |  | 1 | - |  | - |  |
|  |  | 2 | - |  | - |  |
|  |  | 3 | - |  | - |  |
|  |  | 4 | - |  | - |  |
|  |  | 5 | - |  | - |  |
|  | Extent of immuno-positive cells within olfactory epithelium | 0 | 5 (#2-#6) | Median score:  0 | 3 (#7-#9) | Median score:  0 |
|  |  | 1 | 1 (#1) |  | - |  |
|  |  | 2 | - |  | - |  |
|  |  | 3 | - |  | - |  |
|  |  | 4 | - |  | - |  |
|  |  | 5 | - |  | - |  |
|  | Immuno- positive cells within intraluminal exudate | 0 | 6 (#1-#6) | Median score:  0 | 3 (#7-#9) | Median score:  0 |
|  |  | 1 | - |  | - |  |
|  | Subepithelial immuno- positive cells | 0 | 5 (#2-#6) | Median score:  0 | 3 (#7-#9) | Median score:  0 |
|  |  | 1 | 1 (#1) |  | - |  |
| Median of total IHC nose score (maximum attainable score: 12) | | | 0 | | 0 | |
| Liver | Extent of immuno-positive cells (maximum attainable score: 4) | 0 | 2 (#3, #6) | Median score:  1 | 2 (#7, #9) | Median score: 0 |
|  |  | 1 | 3 (#2, #4, #5) |  | 1 (#8) |  |
|  |  | 2 | - |  | - |  |
|  |  | 3 | 1 (#1) |  | - |  |
|  |  | 4 | - |  | - |  |
| Kidney | Extent of immuno-positive cells (maximum attainable score: 4) | 0 | 4 (#2, #4-#6) | Median score:  0 | 3 (#7-#9) | Median score:  0 |
|  |  | 1 | 2 (#1, #3) |  | - |  |
|  |  | 2 | - |  | - |  |
|  |  | 3 | - |  | - |  |
|  |  | 4 | - |  | - |  |
| Spleen | Extent of immuno-positive cells (maximum attainable score: 4) | 0 | 2 (#3, #4) | Median score:  1 | 3 (#7-#9) | Median score: 0 |
|  |  | 1 | 3 (#2, #5, #6) |  | - |  |
|  |  | 2 | 1 (#1) |  | - |  |
|  |  | 3 | - |  | - |  |
|  |  | 4 | - |  | - |  |
| Intestine | Extent of immuno-positive cells (maximum attainable score: 4) | 0 | 6 (#1-#6) | Median score:  0 | 3 (#7-#9) | Median score:  0 |
|  |  | 1 | - |  | - |  |
|  |  | 2 | - |  | - |  |
|  |  | 3 | - |  | - |  |
|  |  | 4 | - |  | - |  |
| Pancreas | Extent of immuno-positive cells (maximum attainable score: 4) | 0 | 6 (#1-#6) | Median score:  0 | 3 (#7-#9) | Median score:  0 |
|  |  | 1 | - |  | - |  |
|  |  | 2 | - |  | - |  |
|  |  | 3 | - |  | - |  |
|  |  | 4 | - |  | - |  |
| Adrenals* | Extent of immuno-positive cells (maximum attainable score: 4) | 0 | 5 (#1, #3-#6) | Median score:  0 | 3 (#7-#9) | Median score:  0 |
|  |  | 1 | - |  | - |  |
|  |  | 2 | - |  | - |  |
|  |  | 3 | - |  | - |  |
|  |  | 4 | - |  | - |  |
| Brain | Extent of immuno-positive cells (maximum attainable score: 4) | 0 | 5 (#1-#4, #6) | Median score:  0 | 3 (#7-#9) | Median score:  0 |
|  |  | 1 | 1 (#5) |  | - |  |
|  |  | 2 | - |  | - |  |
|  |  | 3 | - |  | - |  |
|  |  | 4 | - |  | - |  |

1. **Protein sequence homology data**

**Table S4.** Protein sequence homology of A/grey seal/Netherlands/302603/2023 genome segments

| **Isolate name** | **Protein** | **Amino acid identity** | **Reference strain*** | **Segment ID** |
| --- | --- | --- | --- | --- |
| A/grey seal/Netherlands/302603/2023 (EPI_ISL_17672782) | PB2 | 100% | A/grey seal/Netherlands/30448/2023 (A/H5N1) protein PB2 | EPI2557224 |
|  | PB1 | 99% | A/Domestic_Goose/England/161197/2022 (A/H5N1) protein PB1 | EPI2278015 |
|  | PA | 99% | A/Eurasian_sparrowhawk/Norway/2023-07-18/2023 (A/H5N1) protein PA | EPI3104463 |
|  | HA | 99% | A/chicken/England/152082/2022 (A/H5N1) protein HA | EPI2475293 |
|  | NP | 99% | A/Ardea alba/Slovenia/PER142-24MAZ_24VIR9358-8/2024 (A/H5N1) protein NP | EPI2557212 |
|  | NA | 99% | A/common_buzzard/England/255858/2022 (A/H5N1) protein NA | EPI2300561 |
|  | M1 | 100% | A/Gallus_gallus/Belgium/02296_0002/2025 (A/H5N1) protein M1 | EPI4106237 |
|  | M2 | 100% | A/wood duck/Tennessee/79/2024 (A/H5N1) protein M2 | EPI4100732 |
|  | NS1 | 99% | A/Mallard/Netherlands/15/2022 (A/H5N1) protein NS1 | EPI2197827 |
| *first result of amino acid sequence appearing in the GISAID blast search. | | | |  |

**Supplementary Appendix references**

1. Mirolo M, Pohlmann A, Ahrens AK, et al. Highly pathogenic avian influenza A virus (HPAIV) H5N1 infection in two European grey seals (Halichoerus grypus) with encephalitis. Emerg Microbes Infect. 2023 Dec;12(2):e2257810.
2. Pohlmann A, King J, Fusaro A, et al. Has epizootic become enzootic? Evidence for a fundamental change in the infection dynamics of highly pathogenic avian influenza in Europe, 2021. mBio. 2022 Aug 30;13(4): e0060922.
3. Qasem L, Cardew A, Wilson A, et al. Tri-axial dynamic acceleration as a proxy for animal energy expenditure; should we be summing values or calculating the vector? PLoS One. 2012;7(2):e31187.
4. Simon M, Johnson M, Madsen PT. Keeping momentum with a mouthful of water: behavior and kinematics of humpback whale lunge feeding. J Exp Biol. 2012 Nov 1;215(Pt 21):3786-98.
5. Guilfoyle, K., Mirolo, M., van Amerongen, G., van der Net, G., Lombardo, M. S., Störk, T., … Osterhaus, A. (2024). Susceptibility of calf lung slice cultures to H5N1 influenza virus. Emerging Microbes & Infections, 14(1). <https://doi.org/10.1080/22221751.2024.2432368>
6. Ward CL, Dempsey MH, Ring CJ, et al. Design and performance testing of quantitative real time PCR assays for influenza A and B viral load measurement. J Clin Virol. 2004 Mar;29(3):179-88.

**Alt Text Supplementary Figures and Tables**

**Table S1:** Table of data showing respiratory rate measurement counts from ferrets infected with A/grey seal/NL/2023 or A/Indo/2005 viruses in which the virus strain, logger ID, and number of successful recordings are illustrated.

**Figure S1:** Three accelerometery data plots depicting the respiration signals per minute from ferret #2 before and after infection with A/grey seal/NL/2023 H5N1 influenza virus, illustrating increased respiration signals over time.

**Figure S2:** Six line-charts showing changes in respiration rate and body temperature of ferrets before and after virus infection, illustrating mean and standard deviations and highlighting hyperventilation and hypothermia upon A/grey seal/NL/2023 infection.

**Figure S3:** Line chart showing the amplification efficiency of influenza A matrix protein gene RT-qPCR assay, with Cq values plotted against RNA input concentrations and linear regression analysis demonstrating the assay efficiency across ten 10-fold dilutions.

**Table S2:** Detailed pathohistochemical scoring values in lung, trachea, nose, liver, kidney, spleen, intestine pancreas, adrenals and brain tissue of virus-infected ferrets assigned based on levels of inflammation, necrosis, edema, and epithelial changes.

**Table S3:** Detailed immunohistochemical scoring values in lung, trachea, nose, liver, kidney, spleen, intestine pancreas, adrenals and brain tissue of virus-infected ferrets assigned based on the numbers of influenza virus antigen positive cells.

**Table S4:** Table showing protein sequence homology of A/grey seal/Netherlands/302603/2023 H5N1 virus genome segments containing the isolate name, name of each protein, accession number, amino acid identity to the reference strain.
